# Targeting Probiotics in Rheumatoid Arthritis

**DOI:** 10.3390/nu13103376

**Published:** 2021-09-26

**Authors:** Simona Gabriela Bungau, Tapan Behl, Anuja Singh, Aayush Sehgal, Sukhbir Singh, Sridevi Chigurupati, Shantini Vijayabalan, Suprava Das, Vasanth Raj Palanimuthu

**Affiliations:** 1 Department of Pharmacy, Faculty of Medicine and Pharmacy, University of Oradea, 410028 Oradea, Romania; 2 Doctoral Scool of Biological and Biomedical Sciences, University of Oradea, 410073 Oradea, Romania; 3 Chitkara College of Pharmacy, Chitkara University, Rajpura 140401, Punjab, India; anuja.sansanwal@gmail.com (A.S.); aayushsehgal00@gmail.com (A.S.); sukhbir.singh@chitkara.edu.in (S.S.); 4 Department of Medicinal Chemistry and Pharmacognosy, College of Pharmacy, Qassim University, Buraidah 52571, Saudi Arabia; sridevi.phd@gmail.com; 5 School of Pharmacy, Faculty of Health and Medical Sciences, Taylor’s University, Subang Jaya 47500, Malaysia; v.shantini92@gmail.com; 6 Deprtment of Pharmacology, Faculty of Medicine, AIMST University, Semeling, Bedong 08100, Malaysia; dr.suprava.das@gmail.com; 7 Department of Pharmaceutical Biotechnology, JSS College of Pharmacy, JSS Academy of Higher Education & Research, Ooty 643001, Tamilnadu, India; vasanth1780@gmail.com

**Keywords:** probiotics, gut microbiota, *Lactobacillus*, *Bifidobacterium*, rheumatoid arthritis

## Abstract

Rheumatoid arthritis (RA) is a progressive inflammatory disorder characterized by swollen joints, discomfort, tightness, bone degeneration and frailty. Genetic, agamogenetic and sex-specific variables, *Prevotella*, diet, oral health and gut microbiota imbalance are all likely causes of the onset or development of RA, perhaps the specific pathways remain unknown. *Lactobacillus* spp. probiotics are often utilized as relief or dietary supplements to treat bowel diseases, build a strong immune system and sustain the immune system. At present, the action mechanism of *Lactobacillus* spp. towards RA remains unknown. Therefore, researchers conclude the latest analysis to effectively comprehend the ultimate pathogenicity of rheumatoid arthritis, as well as the functions of probiotics, specifically *Lactobacillus casei* or *Lactobacillus acidophilus*, in the treatment of RA in therapeutic and diagnostic reports. RA is a chronic inflammation immunological illness wherein the gut microbiota is affected. Probiotics are organisms that can regulate gut microbiota, which may assist to relieve RA manifestations. Over the last two decades, there has been a surge in the use of probiotics. However, just a few research have considered the effect of probiotic administration on the treatment and prevention of arthritis. Randomized regulated experimental trials have shown that particular probiotics supplement has anti-inflammatory benefits, helps people with RA enhance daily activities and alleviates symptoms. As a result, utilizing probiotic microorganisms as therapeutics could be a potential possibility for arthritis treatment. This review highlights the known data on the therapeutic and preventative effects of probiotics in RA, as well as their interactions.

## 1. Introduction

Rheumatoid arthritis (RA) is a chronic autoimmune disease characterized by swelling, nerve pain, stiffening, decreased functioning, fractures and cartilage deterioration, all of which contribute to dysfunction [1,2]. RA affects 1% of individuals aging 20–40 years worldwide, and that it’s widespread in people over the age of 75 and older [3]. Women are predisposed to RA at a higher rate [4]. Diabetes, heart conditions, nephritis, lung cancer/chronic obstructive pulmonary disease (COPD), psychological problems, hypertension, asthmatic and cancer are prominent in RA patients, resulting in an increased death rate [5].

Nonsteroidal anti-inflammatory medicines (NSAIDs) are currently used to treat RA because they limit prostaglandin synthesis by suppressing the cyclooxygenase enzymes 1 and 2 (COX-1 and COX-2) and hence reduce redness in the body. Meloxicam, diclofenac, indomethacin, ketoprofen, naproxen ibuprofen and other medications fall within this category [6]. Aside from NSAIDs, disease-modifying anti-rheumatic drugs (DMARDs) such as methotrexate are utilized as first-line RA medication, however, one-fifth to almost one-third of patients with RA were unable to complete medication for more than 12 months due to potentially negative responses. The most prevalent outcomes of the medication are gastrointestinal toxicity (nausea in 20–70% of RA patients). Hydroxychloroquine and chloroquine are antimalarial medicines that have been used to treat RA. Individuals with renal and hepatic impairments may experience toxic effects from co-administration of these drugs because they are weaker bases and have a prolonged shelf life (50 days) in the blood [7]. 

It has already been observed that individuals with RA have enhanced gut permeation due to gastrointestinal (GI) tract inflammation that causes food antigens and highly dangerous microorganisms to travel through the blood as an outcome. Antibodies to such antigens were found to be increased in individuals with RA [8], resulting in the immunological complex in ducts feeding the joint [9]. A meta-analysis indicated that fasting and a vegan diet had a statistical and therapeutically beneficial effect in patients with RA whilst the study revealed how this method affected the microbiome [10].

Probiotics are described as “live microorganisms that bestow health benefits when provided in suitable amounts” [11]. Although it is unclear how well probiotics act, indications of published data are suggesting that they lower gut permeability and influence the immune system as well. This regulation could occur by boosting distal secretory immunoglobulin A (IgA) immunological reactions to pathogens [12], limiting bacterial strains’ proliferation, or decreasing activating immunological components as tumor necrosis factor alfa (TNF-α) [13]. Probiotics are microorganisms that, when given in sufficient proportions, impart a therapeutic effect upon the individual; these advantages vary depending on the strain. The utilization of probiotics to reduce the risk of developing arthritis is relatively unknown, however, some researchers have suggested that it may be beneficial. Oral probiotic treatment has been proven in animal models to reduce the prognostic value of arthritic processes [14]. Gut bacteria may play a role in the development of arthritis, according to research. Researchers have been attracted by the link between the microbiome and immune-mediated diseases. Microbiota refers to the billions of commensal microorganisms that live on human epithelial surfaces such as the gut, respiratory system and skin (bacteria, phage, viruses and unicellular eukaryotes). However, bacterial microbes and their involvement in regulating immune responses have recently gained a lot of interest due to their role in modulating immune function, even though the relevance of viruses, archaea and unicellular eukaryotes is still largely understood.

Symbiotics are formed of living microbes when provided in proper concentrations could enhance the health of the host. These are produced by the interaction of probiotics or prebiotics, being supplements that combine pro-biotics (good bacteria for the gut) with prebiotics (non-digestible fibers that help these bacteria grow). A symbiotic substance has a favorable effect on the host by selectively encouraging the growth and/or activating the metabolism of one or a small number of health-promoting bacteria (this phrase should only be used for items that include prebiotic compounds that favor the growth of probiotic organisms). Symbiotic were created to help probiotics survive any problems that might arise. The rationale for utilizing symbiotic appears to be based on observations that it shows a boost in probiotic bacteria viability throughout passage via the upper digestive tract. Improved colon implantation and a stimulating influence on the growth of probiotics and ubiquitous bacteria was found [15].

It has also been established that the combination of methotrexate and probiotics (*Enterococcus faecium*) lowers therapeutic measures compared to animals receiving alone methotrexate treatment [16,17]. The imbalanced GI microflora in individuals with ankylosing spondylitis suggests dislocation of infections (in this case sulphate-reducing bacteria) and inclusion of commensals such as *Lactobacilli* and *Bifidobacteria* could confer significant metabolic and symptomatic assistance. A study in Lewis rats utilizing a RA model found that *Lactobacillus rhamnosus* GG intake can give some anatomical and functional relief in swelling at joint locations [18]. The clinical picture of RA is produced by the interaction of antibodies and immunological mechanisms involved in the host immune response. There are immune cells that penetrate the joint tissue of RA patients. These cells produce cytokines that promote inflammation and eventually tissue degeneration, leading to joint destruction. Modification of GM may regulate the gut immunological tolerance mechanism. Due to its influence on the quantity and function of colonic regulatory T cells (Tregs), GM modification may help modulate gut immunological tolerance [19,20].

There is some evidence to suggest that probiotics help lower blood levels of total cholesterol (TC) and low-density lipoprotein cholesterol (HDL-C). In animal studies, *Lactobacillus casei* strains were discovered to have hypolipidemic effects. Dendritic cell toll-like receptors (TLR-7 and TLR-9), as well as the production of major histocompatibility complex (MHC) class II molecules, are inhibited by the medicines, which modulate B-cell-mediated release of inflammatory cytokines—interleukins (IL-1, IL-6, etc.). Administering *L. casei* fermented milk to rats on a high cholesterol diet reduced cholesterol accumulation; HDL-C was considerably enhanced in the probiotic cluster, whereas serum triglyceride (TG) was infinitesimally reduced [21]. Low-fat milk cultured with *L. casei* Shirota in rodents lowered plasma TG on both a higher and lower-fat diet. Additionally, when rodents were given solubilized *L. casei ssp*. for 90 days, the resulting reduction in triglycerides was 15 to 25%, depending on the category of skim milk. There was no statistically significant change in RA activity with *L. rhamnosus GG*, while additional participants in the probiotic group informed a progression in subjective well-being when clinical research was significantly underpowered (*n* = 10 patients) and examined solely moderate RA [22]. Since *L. rhamnosus* and *L. reuteri* have pro-inflammatory capabilities, such variants should be studied in the treatment of RA. Pro-inflammatory characteristics of the probiotics *L. rhamnosus GR-1* and *L. reuteri RC-14* were suggested to allow persons with RA to increase their everyday activities.

According to a survey, consumers are well aware of probiotics and frequently utilize them. Individuals’ probiotic use decisions are heavily influenced by factors such as age, occupation, expenditure and education, as well as lifestyle features such as exercising routinely and reviewing food labels. Most active probiotic users are likely to continue using probiotics, and the usage is expected to rise in future years, according to the research findings. The millennial generation uses probiotics regularly, which means that probiotics with indications of effectiveness have a significant potential for addressing public health challenges in the future. The survey also found among the non-users, the most common reasons for not utilizing probiotics are financial restrictions and a positive perception of one’s health [23]. Users prefer yogurt and shake to other carrier forms of probiotics, regardless of whether they are already utilizing them. Overall, our findings demonstrate that youth are already a significant probiotics market, with room to grow. The availability of probiotics that fit the desires and expectations of consumers may result in greater probiotic consumption by millennials, which could have a favorable impact on public health [24]. Increased use of probiotics by young adults could result from the availability of probiotics that fit their preferences and expectations, which could have a favorable impact on public health, healthcare spending and the general economy. The capacity of probiotics to solve public health challenges will also be determined by how health care practitioners and the probiotic sector take advantage of factors that influence consumers’ level of awareness and use [25]. 

We chose this topic starting from the observation of the recent and more and more accentuated attention of the specialists focused on the attempt to use probiotics in the treatment of various diseases. Thus, the purpose of this study was to evaluate the possibility of using probiotics and their usefulness as adjuvant therapy, in addition to classical therapeutic medication, to optimize the management of the health status of patients with RA [26]. Thus, we’ve succeeded in providing a solid framework for information on this topic after searching the most popular medical databases (PubMed, EMBASE, Cochrane Library, etc.), helping readers and future studies in the field in better understanding the relationship of probiotics with RA.

## 2. An Inflammatory Pathway in Rheumatoid Arthritis

It is categorized by enhanced fluid accumulation, incendiary cell differentiation of the joints, and stimulates the release of immune cells (IL-1, IL-17 and TNF-α) [27]. TNF-α and IL-6 antagonists have been used in experiments to be beneficial against RA [28]. Decreased development of immunoregulatory mediators such as IL-11, IL-13 and IL-10 leads to the immune response [29]. Chronic inflammatory disorders such as RA are caused by an elevated percentage of pro-inflammatory to anti-inflammatory cytokines, which is induced by helper T cell type 1 (Th1) [30]. TNF-α, IL-17A, IL-17F and IFN (-interferon) are produced by helper T17 (Th17) cells (which protect from foreign microorganisms) and contribute to the pathophysiology of RA [31]. Several receptors that identify pathogen-virulence factors, such as TLRs, regulate RA by activating host-defense mechanisms and maintaining adaptive inflammatory reactions. TLRs control nuclear factor kappa-B ligand (NF-B), osteoclast formation and promote the elevated generation of TNF-α, IL-6, IL-12, IL-18 and a variety of other proinflammatory cytokines. TLRs are found in the cartilaginous fluid, where they generate inflammation that results in swollen joints, discomfort, rigidity and bone and cartilage deterioration. TNF-α and IL-1 levels rise in the blood and synovial tissues, speeding up the stimulation of matrix metalloproteinase (MMP) enzymes (e.g., MMP-1, MMP-9 and MMP-13), which could disintegrate extracellular matrix and joint cartilage components. RA onset and development are linked to several hereditary, ecological and socioeconomic variables. Various inflammatory states and outcomes in RA are caused by extra fat and limited diet, tobacco, intestinal microbiota, periodontal disorders and bacterial or viral infections [32,33]. A rising number of studies have shown that nutrition has a significant impact on the microbiota’s makeup and function, with crucial implications for human health and illness. Throughout the last few centuries, people’s eating patterns have evolved dramatically, giving rise to a whole new dietary category. It’s important to note that substantial reductions in dietary fiber intake have characterized a “fiber gap” or inability to deliver enough nutrients to the microbiota, resulting in the decrease of its activities, which has important pathophysiological implications. The microbiota’s composition is influenced by the socioeconomic background and lifestyle because of dietary patterns [34].

## 3. Probiotics and Their Interventions in RA

Probiotics are supposed to benefit health by boosting the useful bacteria in the stomach (gut microbiota). In theory, some of the bacteria found in our bodies, such as *Lactobacillus* and *Bifidobacterium* types, are also available in probiotic supplements or foods. Everyone’s body is home to a unique variety of microorganisms (in the gut, on the skin and in the mouth, for instance) [35].

Diet, environment, drugs and experiences all have an impact on these communities throughout time. Scientists are now discovering that they may have an impact on many elements of human functionality. There is a broad consensus that gut microorganisms have a larger role in human health than we previously believed and many of the healthy microbes work together to keep the harmful bacteria in control, which is advantageous to your general health. Experts are uncovering also that gut microbiota is critical to human survival, for instance, by assisting the immune cells in determining whether it is safe or threat and assisting the digestive tract in extracting necessary nutrition. Most of these microbes seem beneficial, some may be harmful, some tend to be impartial and others are equally healthy and unhealthy based on the circumstances. There seems to be an intricate association between gut microbiota and disorder. Whenever there is a chronic condition that affects the gastrointestinal tract, including immune-mediated forms of arthritis, probiotics have the potential to treat it [36,37].

Probiotics appear to function in three ways:

- Keeping an equilibrium of “beneficial” and “harmful” bacteria in the body;
- Diminishing harmful bacteria that can cause allergies and ailments;
- Renewing beneficial bacteria that have been lost post sickness.

There is increasing understanding that *Lactobacillus* and *Bifidobacterium*, probably one of the best probiotics, may aid the system in a variety of ways, including:

- Accelerating the treatment of some intestinal infections;
- Assisting in the reduction of gas and bloating;
- Preventing or minimizing the severity of colds and flu;
- Improving blood pressure;
- Relieving symptoms of inflammatory bowel illnesses such as Crohn’s disease and ulcerative colitis.

Probiotics may be highly beneficial if you have inflammatory arthritis [38,39]. The good bacteria seem to provide an impact on the treatment, lowering common inflammatory indicators such as C-reactive protein (CRP). Individuals with RA have also demonstrated inflammation in the intestinal wall which leads to greater intestinal motility [40]. This allows specific bacteria to pass the gut barrier, enter the circulation of the blood and produce an infection. Probiotics can help reduce inflammation due to increased intestinal permeability. Individuals with RA and allergic diseases have a changed gut microflora, showing that the normal intestinal microflora is ecologically important that reacts to the swelling in the stomach and around the human body. The rationale for probiotic therapy is to normalize the qualities of unbalanced native microbiota by using selected strains of healthy gut microflora. Generalization of enhanced gastrointestinal permeability and changed gut microecology, strengthening of the intestine’s immune barrier activities and relief of the intestinal immune response are all signs of probiotic effectiveness. Clinical ailments requiring poor mucosal barrier properties, notably viral and proinflammatory disorders, are identified as potential targets for probiotic therapy [41,42]. 

A nutritious diet helps maintain strong intestinal barriers and a highly combated immune system, healthy diets and probiotic supplementation can complement each other to keep joints healthy and the rest of the body strong. 46 patients with RA were categorized into two groups in a recent report in the journal. A group was given *Lactobacillus casei* supplements daily, while the other was given a placebo [43]. During an eight-week term, multiple markers of inflammation were much reduced in the probiotic group, prompting researchers to conclude that, while more research is needed to validate the findings, probiotic administration may be a useful supplementary therapy for individuals with RA. 

Another study, published, discovered that gut bacteria in mice could be analysed to determine which animals were more prone to developing RA and collagen-induced arthritis (CIA)—the rodent version of osteoarthritis [44,45]. This prompted researchers to speculate that the gut microbiome could be a possible predictor of arthritis susceptibility.

### 3.1. Probiotics Selection

Probiotics and their therapeutic benefits on many health issues are still unknown. Furthermore, supplements are not adequately monitored, so there is no assurance that they include the same forms of probiotics that have been clinically confirmed to be helpful [46]. According to a recent investigation, 30% of probiotic pills did not contain the levels of beneficial organisms claimed on their labels. There is no meaningful consumer advice for probiotic supplements, people must evaluate the type and dosage and compare that to medical research to see whether it will help—but most people will not take the time to do that, so there will be a lot of trial and error [47,48]. Check for medications with a USP label, that signifies that the components have been confirmed by an external company, and consult your doctor before beginning any supplementation; some could interfere with existing prescriptions or cause other unforeseen effects [49]. A variety of assertions are made about probiotics’ methods of action and “vital” qualities based on conjecture rather than scientific research. Examples include the requirement that the probiotics be able to attach themselves to gut cells. Even though much of the evidence for adhesion comes from in vitro studies, that have restricted prediction for in vivo studies, it should be harmonized with the fact that, in general, probiotics are only present for a short period after feeding has ceased inside the host body. As a result of this, some strains of *Bifidobacterium animalis* that are not generally isolated from humans are efficient probiotics. A common claim is that probiotics help to maintain healthy microbiota. There is some evidence that probiotics can affect the populations or activity of colonizing microorganisms (lactobacilli or bifidobacteria) levels in the faces are commonly correlated with improved balance. When the microbiota is disturbed (e.g., by antibiotics or disease), probiotics may help restore normalcy, or at the very least, limit the degree of change induced by such challenges. This activity lends greater credence to the idea that probiotics help improve microbial balance [50,51].

In humans, a probiotic-enhanced recovery to baseline levels following antibiotic usage has been measured in a few investigations. Further research on the hypothesis of probiotic-induced better microbiota balance might be beneficial. When choosing probiotic strains, it’s crucial to consider both efficacy and scientific functionality. It would be a difficult undertaking to establish a list of qualities required for all probiotic actions because the spectrum of targets for in vivo function is so extensive, including oral, gastric, pulmonary, gastrointestinal, genital and immunological functions [52]. Fundamental strain identification and taxonomic characterization should be carried out first, followed by testing with established assays in lab animals and controlled research in the targeted system. In vitro studies are commonly used, however, they have yet to be proven to predict in vivo function. Scientific resilience, such as the strain’s ability to grow in large numbers, concentrate, stabilize and combine into a finished product with good sensory qualities, if appropriate, must also be assessed, as must the strain’s physiological and genetic stability throughout the product’s shelf life and at the functional center in its host [53]. 

Certain lactobacilli strains have been demonstrated in studies to be useful in reducing antibiotic-associated infection, many of its species being frequently chosen as probiotics because they possess some important characteristics, including: a high tolerance to acid and bile; the ability to tolerate low pH, gastric juice; the ability to inhibit potentially pathogenic species; the ability to resist antibiotics; the ability to produce exopolysaccharides; and the ability to remove cholesterol. *Lactobacillus rhamnosus* CRL1505 has also been shown to be beneficial in lowering viral-associated lung damage by regulating immune-coagulative responses and eliminating respiratory viruses [54,55]. Due to their extensive spectrum of bile salt resistance mechanisms, *Bifidobacterium* strains are commonly used as probiotic bacteria. This is important because the therapeutic effects of probiotic bacteria should arise in the vicinity of such a biological fluid. However, research has revealed that the bile threshold varies by strain and that bile-sensitive wild-type bacteria and Lactobacillus strains can respond to the proximity of bile salts by culturing and progressively raising bile salt concentration. *Bifidobacterium infantis*, *B. adolescentis*, *B. animalis* subsp. *animalis*, *B. animalis* subsp. *lactis*, *B. bifidum, B. longum* and *B. breve* are all regarded as essential probiotics [56]. 

### 3.2. Role of Diet in RA

A well-balanced diet is one that contains a variety of meals in specific quantities and proportions to meet the body’s needs for carbohydrates, proteins, minerals, vitamins and other nutrients, while also reserving a small amount of nutrients for when the body is at its leanest. A healthy, well-balanced diet gives you the strength you need to be active all day and reducing your risk of disease caused by poor diet, such as some heart disease and diabetes [57].

However, the effect of dietary changes in RA is not fully recognized. Several types of research have been conducted in an attempt to fill these information gaps. For the prevention and treatment of RA, intestinal microbial changes are being investigated. Antioxidants, lactobacilli, fibber and changes in the intestinal flora may be responsible for some of the benefits of a vegan diet. Dietary omega-3 polyunsaturated fatty acids and vitamins, as well as gut microbiota, influence the anti-inflammatory effects of the Mediterranean diet. Diets that are low in carbohydrates and high in vitamins and minerals have been linked to some benefits in RA, however, the data is limited. A diet of long-chain polyunsaturated fatty acids (PUFAs) such as those found in fish and other sources is protective against the development of RA. For people with rheumatoid arthritis (RA), the advantages of flavonoids, antioxidant supplements and fasting, probiotics remain unclear. RA disease activity has been proven to be reduced when vitamin D is consumed. RA-specific dietary advice should consider the role of supplements such as fish oils and vitamin D in future research. Changes in diet can affect the human gut microbiome, causing local inflammation and increased permeability. This can result in the transmission of pro-inflammatory cells and cytokines throughout the body, causing inflammation in distant areas such as the joints. Diet and weight loss have been shown to reduce the disease load in RA. Disease-modifying medications and dietary adjustments that alter the microbial environment appear to regulate RA’s disease process and progression [58]. 

Numerous studies have shown that nutrition can influence RA’s clinical course through a variety of pathways, and this is a major concern for both patients and physicians. Rheumatoid patients frequently seek dietary advice and engage in experimental diets in the hopes of reducing their symptoms and slowing the progression of their condition. Results can be mixed at best, therefore knowledge in dietary behavior is needed to accompany modern pharmacological treatment. According to research, eating fruits, vegetables and whole grains lowers one’s chance of acquiring a variety of infectious conditions, whereas consuming red and processed meats, simple carbs and added sugars raise one’s risk. Lacto-ovo-vegetarian or vegan diets, the MedDiet, elemental eating plans and exclusion diets are among the most frequently recommended dietary approaches [59]. 

## 4. Mechanism of Action of Probiotics in Rheumatoid Arthritis

**Figure 1:**
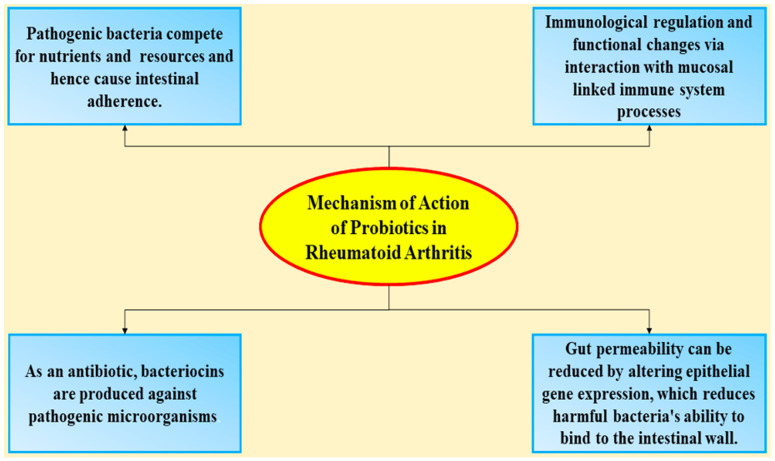
Probiotics in rheumatoid arthritis.

While probiotics’ local impacts on gut health were well established, the mechanisms behind their widespread anti-inflammatory and immunomodulatory ability remain largely unknown. As well as having a local effect on gut health, SCFAs are known to have a significant influence on the external inflammatory process and oxidative stress through the control of different innate immune activities [60,61]. Butyrate has been shown to inhibit antigen-induced inflammation in rodents by influencing the B lymphocyte’s cellular proliferation. For example, it suppresses antigen-specific B lymphocytes and plasmablastic proliferation as well as innate natural killer T (NKT) cell cytokine production, a cell subgroup implicated in joint inflammation and tissue degradation [62]. The mechanism of probiotics’ action in RA is schematized in Figure 1.

In a separate rodent model of RA, there was an increase in polarity towards that T regulatory (Treg) phenotype with a decrease in Th17 cells, as well as suppression of pro-inflammatory cytokines [63]. Other mechanisms, in addition to SCFA, could also be postulated to elucidate the therapeutic activity of probiotics in rheumatic illnesses. The control of T helper and T reg cell activities, as well as the generation of immunological tolerance, appear to be the most important. *L. casei* strain Shirota has been demonstrated to inhibit the occurrence of autoimmune illnesses by altering the production of cytokines from antigen-presenting cells and, as a result, lymphocyte differentiation toward effector T cell-specific subsets [64]. It is of great significance since the Th1-Th17 response is crucial in the early stages of the development of autoimmune illnesses, particularly inflammatory rheumatic disorders. In experimental immunological illnesses such as RA, probiotic microorganisms have been demonstrated to generate a Treg immune response [65]. They have been demonstrated, in particular, to promote the transformation of T cells into Tregs via the forkhead box transcription factor FOXP3 and to enhance the suppressive function of pre-existing Tregs [66].

The decrease in antibody immune response has also been linked to an increase in FoxP3 positive Treg cells. In a certain approach, a decrease in antibody immune response has been linked to an increase in FoxP3 positive Treg cells, an increase in anti-inflammatory cytokines, and a decrease in pro-inflammatory cytokines [67]. In an animal model of collagen-induced arthritis (CIA), oral treatment of *L. casei* Shirota reduced the abnormal antibody production produced by type 2 collagen immunization. An increase in anti-inflammatory cytokines, and a decrease in pro-inflammatory cytokines, are all signs of FoxP3-positive Treg cells. SCFA, histamine and adenosine are examples of probiotic-modulated local and systemic metabolites with anti-inflammatory and antibacterial properties [68]. A fundamental mechanism by which probiotics compete in this environment is selective alienation, by which they attach to the gut wall and prevent pathogens from entering the lamina propria thereafter [69]. SCFAs have an indirect anti-inflammatory effect through enhancing gut barrier integrity. For example, butyrate inhibits histone deacetylase and modulates the activation of multiple pro-inflammatory genes, leading to the division and growth of Tregs and controlling cytokine release [70]. Assessing that a dysbalanced gut microbiome is a critical trigger in the pathophysiology of RA, clinical interest in probiotics to rectify gut dysbiosis and reduce the inflammatory cytokine cascade has evolved. SCFAs have anti-inflammatory characteristics because they regulate various leukocyte functions, including the generation of inflammatory cytokines, chemokines, such as Tumor necrosis factor, IL-10 and IL-2 [71]. The metabolic products of probiotic bacteria and their adaptability to the gut mucosa and sensitivity to pathogenic microbial invasion, attachment and movement can be maintained by using probiotics. With these features, it may be possible to reduce gut wall permeability, which is thought to be a contributing factor to inflammatory arthritis. Transmigration of cytokines and expansion of regulatory T cells (Tregs) at the site of inflammation is a common process. SCFA, a probiotic metabolic product, affects immunological reaction and increased inflammation by influencing immune cell activity [72].

**Figure 2:**
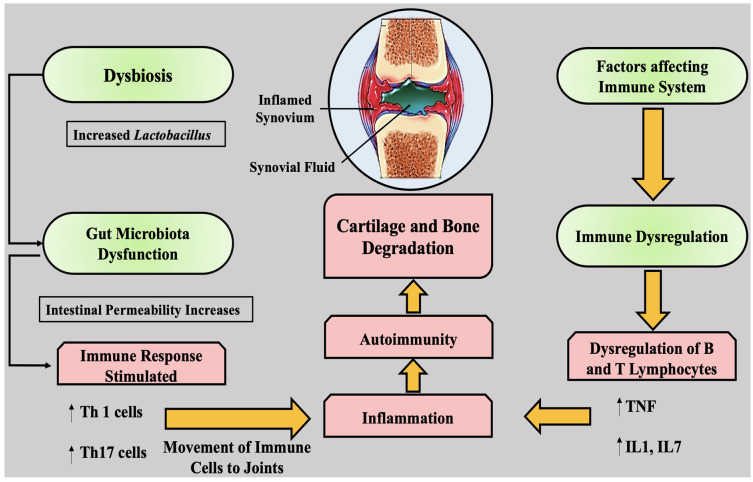
Factors affecting the immune system. Th, T helper; TNF, tumor necrosis factor; IL, interleukin; ↑, increasing/activating/enhancing; ↓, decreasing/inhibiting/reducing.

A signaling cascade that results from the attachment of probiotic bacteria to epithelial cells may modulate the immune system. According to studies conducted in humans, this activity could reduce oxidative stress and minimize cardiac problems linked with chronic inflammation in RA. Factors affecting the immune system are summarised in Figure 2.

## 5. Rheumatoid Arthritis and Gut Microbiota

The gut microbiota is gained significant focus as a key contributor to the development of a range of disorders [44,45]. As a result of several powerful technologies, researchers can now conduct extensive investigations of microbial populations, showing that they could influence the pathophysiology of inflammation conditions such as RA. Studies have shown that the microbiome influences central immune response in a variety of ways, including changes in bacterial composition, enhanced gut porosity and impact on T-cell pathogenesis. Bacteria and bacterial cells make up approximately 1000 types of bacteria in the human gut and bacteria cells.

The “microbiome” refers to the collection of genes found in the microbiota. The gut microbiome is a massive source of antigenic diversity. From the other perspective, it is postulated that immunological responses to self-antigens represent a key development event in JIA [46,47]. Several groups of effector T cells play a role in juvenile idiopathic arthritis (JIA) pathogenesis. The increased presence of regulatory T lymphocytes in the site of the inflammation (Tregs) in the JIA was also involved. Given the interaction of the gut microbiota with T-cell development, a role for the microbiome in JIA pathogenesis is feasible.

Only laboratory animals have been used to study how the gut microbiome supports the body’s immune growth [48]. Intestinal mucosal inflammatory response stops growing in germ-free mice, according to studies. Th17 cells and Tregs in the colonic basal lamina are declining, whereas Th17 cells are increasing in the intestinal wall basal lamina [73]. These germ-free mouse tests indicated that the gut microbiome influences the intestinal immune system. The K/BxN spontaneous mouse model of arthritis was one of the first to show that symbiotic microbes affect systemic autoimmunity. To prevent arthritis, K/BxN rats are kept in a germ-free environment, which leads to a reduction in the generation of Th17 cells in comparison to traditionally housed K/BxN rodents [74]. A single mono-colonization with the segmentous fibrous bacteria is enough to reactivate rheumatism in this animal, by inducing the production of Th17 cells in the lamina propria, which can then recirculate to the joint and cause arthritis inflammation. The microbiota in the intestine could potentially produce local impacts on vascular permeability and gut resistance. The gastrointestinal mucosa prevents gut microorganisms from reaching lymphoid tissues, preventing dysregulated stimulation of the local inflammatory pathways [75].

Mucin is degraded by several *Bacteroides* species which is an important defence factor. It is fair to believe that mucin breakdown can improve bacteria’s access to the gut immune system, hence fostering an inflammatory response. The number of helpful bacteria in the gastrointestinal systems of people who develop RA is significantly reduced. It might be too early to determine if gut bacteria induce RA, precisely since it is too early to determine whether gut bacteria protect it [76]. We all know that having a healthy gut flora is important for general health. The gut’s walls serve as a habitat for numerous cells of the immune system. These immune cells in the gut play a crucial role as gatekeepers. They have to find and destroy foreign intruders that are riding on our food [77]. The immune system of the intestine, on the other hand, must strike a precise balance between attacking intruders and allowing nutrients into the body [78].

### 5.1. Bacillus coagulans in Rheumatoid Arthritis

Rheumatoid arthritis is regarded as among the most important chronic autoimmune, inflammatory disorders of unknown origin. Research has shown RA sufferers have different gut bacteria than healthy individuals. In RA patients, *Bifidobacterium* species and lactic acid bacteria (LAB) levels are significantly reduced, with varied reports of lower and higher *Clostridium* levels. As a result, dietary management via probiotic remedy may be a noninvasively strategy to controlling the gut microbiota to maintain appropriate GI, downregulating the aberrant inflammatory response and alleviate RA symptoms [75].

It is unclear how probiotics inhibit or cure arthritis; nevertheless, other researchers previously observed a reduction in gut permeability, that relates to immune system modulation. Such regulation activity could be linked to improved localized secretion of IgA inflammatory cells to the pathogen, decreased pathogenic bacterial proliferation and downregulation of immune response components such as IL-12, and TNF-α without altering regulated factors such as IL-10 and tissue growth factor beta (TGF-β) [76]. When certain bacteria and microorganisms are stimulated by probiotics, they boost the health of their hosts. Animal models of RA suggest that taking probiotics orally reduces arthritis severity by decreasing gut permeability [77]. A recent clinical trial assessed the effects of probiotic *L. rhamnosus* oral treatment. The impact of oral administration of probiotic *L. rhamnosus* GR-1 and *Lactobacillus reuteri* RC-14 capsules on RA patients has been assessed in a recent clinical investigation. In comparison to the placebo group, there was a functional enhancement in the probiotic group [79].

*Bacillus probiotics* are a type of probiotic bacteria that live in our intestines. The main distinction between Bacillus organisms and many such various probiotics is that Bacillus microorganisms produce “spores”. Probiotics in spore form are protected from severe stomach acid, allowing them to reach the intestines faster than unprotected types such as lactobacilli or bifidobacteria.

Even though many of these bacteria are found naturally in the stomach, we ingest some via interaction with the soil. Spore organisms can be found in abundance in soil. As individuals eat spores from everyday interactions with soils, they germinate in our small intestine and can survive there for up to three weeks [80]. These subsequently revert to spores and are discharged into the atmosphere until they are picked up by another host. Bacillus probiotics provide us with a lot of health benefits once they’re in our system. *Bacillus coagulans* is one of the most common Bacillus strains. There were no negative side effects noted among those who utilized Bacillus supplements. While we usually associate probiotics with gastrointestinal health and comfort, they also play an important function in our immune system. From within the gut, immune health has an impact on all of our tissues and bodily systems, such as the occurrence and progression of RA [81].

### 5.2. Preclinical Studies on the Effects of Lactobacillus Probiotics on Arthritis

Probiotics are living organisms that are taken orally to improve the microbiota of the host [82]. In conditions such as antibiotic treatment, trauma, NSAIDs and immune diseases such as RA and osteoarthritis, the human gut microbiota could become unbalanced and induce dysbiosis. *Lactobacillus* spp. and *Bifidobacterium* spp. are widespread probiotics organisms, and *Lactobacillus* spp. may thrive in an acidic intestinal surrounding with the support of glucose in the stomach [83]. The effectiveness of probiotics is determined by the host’s microbial strain or pathophysiologic circumstances. In a preclinical model, therapy with *L. casei* or *L. acidophilus* for 28 days inhibited the development of arthritis by reducing cytokines such as IL-17, IL-1, IL-6 and TNF-α [83]. Anti-inflammatory cytokines including Interleukin4 and 10 were similarly elevated in the body fluids after treatment with *Lactobacillus* spp. [84]. Intake of *Lactobacillus acidophilus* and *L. casei* lowers oxidative stress in collagen-induced arthritic rats. In a collagen-induced arthritis model, oral *L. casei* therapy inhibited the cyclooxygenase-2 (COX-2) enzyme and reduced chemokines, leading to anti-inflammatory benefits in rats. The experimental investigation found that intragastric treatment of *L. casei* reduced lymphocyte proliferation and delayed the progress of *Salmonella* enterocolitis-induced arthritis [85]. In rats, treatment with *L. casei* at the beginning of adjuvant-induced arthritis reduced the progression of arthritis in a way it was similar to methotrexate, with stabilization of intestinal microbiota and an increase in *L. acidophilus* number [86,87].

Post *L. casei* therapy in CIA rodents, another survey suggests similar alterations in cytokine profiles, as well as decreased oedema, cartilage degradation and lymphocyte infiltration in joints [88]. By altering the gut microbial community (raising *Lactobacillus* spp.) and preventing CIA in female Wistar rats, various *Lactobacillus* spp., including *L. casei*, *L. reuteri*, *L. fermentum* and *L. rhamnosus* reduced CIA. Numerous *Lactobacillus* spp., particularly *L. casei, L. reuteri*, *L. fermentum* and *L. rhamnosus*, decreased CIA in female rat models by changing the gut microbiome group (rising *Lactobacillus* spp.), prohibiting inflammatory cells, emitting antibodies, antibiotic stimulants, altering Th1/Th17 responses [89,90]. However, on the downside, *L. casei*, *L. salivarius*, *L. ruminis* and *L. iners* have been linked to the pathology of RA with higher populations of these bacteria found in RA patients compared to healthy individuals [91]. 

Additional analysis revealed that giving *L. casei* and *L. acidophilus* orally augmented phagocytic and lymphocytic activities in the intestinal mucosa of albino rats [92]. As a result of the higher abundance of these cells in the intestinal gut, ingestion of *L. casei and L. acidophilus* can occasionally but rarely result in adverse effects. As previously documented, there are differences in the effects of *Lactobacillus* spp., treatment procedures and induction of RA, animal species and measurement parameters. As a result, the findings of the published research are ambiguous, but it can be considered that a moderate population of *L. casei* and *L. acidophilus* has positive benefits.

### 5.3. Clinic Applications of Lactobacillus Probiotics in the Treatment of Rheumatoid Arthritis

*Bacteroides*, *Escherichia* and *Shigella* bacteria were found in higher numbers in the guts of RA patients, while *Lactobacillus* spp. was significantly lower [93]. Essential vitamins of the B vitamins complex—including B3, B5, B6 (pyridoxal phosphate), B7 and B12, as well as folate, tetrahydrofolate and vitamin K, are all provided by well-balanced gut bacteria [94]. 

Inflammatory diseases such as RA have lower plasma folate levels, and prolonged therapy with NSAIDs such as cyclooxygenase blockers inhibited vitamin-B6 metabolism, resulting in lower blood pyrophosphate levels [39,95]. To provide nutritional support and to lower the pH of the intestinal lumen, bacteria release many short-chain fatty acids and vitamins [96]. Assume that there is a random sequence. *Lactobacillus* spp. can also be used as an antibacterial agent against a variety of bacteria [53]. Eight weeks of *L. casei* therapy enhanced RA-related pathological indicators in a randomized clinical trial. Natriuretic and reactive oxygenated species (ROS) levels are elevated, which leads to the breakdown of lipids and various macromolecular components of the affected individual’s matrix [97,98]. *L. casei* in capsules (containing 108 colony-forming units) devouring treatment was found to lower joint swelling, joint problems and inflammatory cytokines in comparison to a placebo-treated control group.

## 6. Probiotics’ Efficacy in the Treatment of Rheumatoid Arthritis

Probiotics has been researched both for human and animal trials to assess possible positive benefits in the prophylaxis and therapeutic approaches for an effective range of autoimmune disorders. Immune-related disorders such as RA, psoriatic arthritis, ulcerative colitis, are demonstrative examples of these conditions. 

Probiotics have many benefits, including the regulation of immune functions, that are usually reliant on the type of probiotic used. Some strains have been shown to stimulate the immunological response, making them advantageous to patients with immunodeficiency [99]. While the origin of RA remains uncertain, new data suggests that microbial dysbiosis at mucosal locations (in the association of stimuli) may play a role in the ailment in biologically susceptible patients. The presence of increased serum-related autoantibodies in early RA despite therapeutically visible synovitis validates such theory, implying that disease develops outside of the joint [100]. 

Dysbiotic gut microbiota was observed in people with initial phase auto-immune provocative RA, which can trigger autoimmune reactions in distant sites such as the joints. Initial mouse models consistently showed a link between intestinal microbiota and systemic immunity, and joint provocative stimulation as well. Researchers in prior probiotic surveys were unable to demonstrate a substantial distinction in RA activity through the use of probiotics, but few observed that supplementation led to enhanced disease severity results in RA patients when compared to placebo in more recent published research [101,102,103]. They discovered lower gut microbial diversity in RA comparison to the control group, that also corresponded with a duration of illness and serum rheumatoid factor levels. *L. casei* 01 supplementations reduced serum high-sensitivity C-reactive protein (hs-CRP) levels, sore and swollen joint counts, and increased global health (GH) score (*p* < 0.05). 

A substantial difference in circulation levels of IL-10, IL-12 and TNF was also seen between the two groups, favoring the probiotic group [104]. The researchers have reported enhanced actual figures of *Lactobacillus salivarius*, *Lactobacillus iners* and *Lactobacillus rimae* in non-treated patients with RA [105]. Researchers anticipate that additional study will be required to identify the microbiome profiles that contribute to RA and the potential for adjuvant probiotic treatment because of these emerging facts.

Recently, more emphasis on the role of nutrition in the pathophysiology of rheumatic disorders has been carried out. Healthy eating habits and the intake of food high in bioactive chemicals, essential fatty acids and antioxidants have been linked to a lower chance of developing certain diseases and a less severe clinical result [106]. 

A substantial relationship between gut microbiota and clinical aspects of patients underlined the prospect of modulating illness progression and presentation through microbiome manipulation. 

Probiotics, live bacteria that, when provided in sufficient proportions, impart a health benefit on the host, may play an emerging role in this context [79]. *Lactobacillus* (L.), *Bifidobacterium* (B.) and *Streptococci* (S.) are the most often employed bacteria, either as a specific organism or in mixed cultures. 

Randomized controlled clinical trials (RCTs) and meta-analyses on infectious illnesses, bacterial diarrhea, inflammatory bowel diseases, abdominal discomfort and colitis have widely identified and supported their health effects [80]. Even though just a few RCTs had researched the impact of probiotics in RA patients, the results have been promising [81]. Despite the small sample size, the medication improved the pain scale, patient systemic risk, self-assessed functionality and serum C-reactive protein (CRP) indicator when relative to the placebo group. These researchers found a significant reduction in serum inflammatory cells however no impact of *L. casei* 01 supplements on the patients’ oxidative condition [82]. 

Only a few fundamental processes underlying probiotics’ positive benefits are currently understood. To establish health claims, molecular characterization of probiotics is necessary due to incomplete information regarding the doses of probiotics required for certain clinical outcomes. Probiotics’ ability to exert their positive benefits is currently only partially understood due to a dearth of direct data. The probiotic interactions among the strains in formulations, which contains a mixture of probiotic strains, have not been explored. Only a small amount of research has been carried out to date on the production process and subsequent formulation, and much more work needs to be carried out to ensure that strains survive the formulation and storage processes. Probiotic(s) that cause harm are uncommon, although the most common adverse effect in GIT, such as bloating. *S. boulardii* and *Lactobacillus GG* have been shown to hasten the onset of problems in certain patient groups, particularly those with impaired immune systems. Since their immune systems are weakened, pregnant women, neonates and the elderly are more susceptible to probiotic infection. Several *Lactobacillus* strains are naturally vancomycin resistant, which raises concerns about the spread of resistance to other harmful organisms in the gut [107]. 

In a group of 60 RA patients, similar outcomes were reported (30 cases and 30 controls). Researchers discovered that probiotic delivery was linked to a substantial increase in insulin sensitivity when subjected to the placebo. On the other hand, no effect was seen on oxidative stress indicators. In this case, 29 patients with RA participated in the sole 12-week trial, which found that combining *L. rhamnosus* GR-1 and *L. reuteri* RC-14 resulted in a marked increase in the (HAQ) level in the probiotics groups however could not therapeutically ameliorate RA [83]. 

Nevertheless, probiotic supplement in RA provides a general advantage to patients, at least in the shorter period. Taking probiotics is a sensible way to promote healthy gut bacteria. Probiotics, on the other hand, are living bacteria. As a result, most probiotics eaten perish in the stomach’s acidity. Ill-advisedly, taking just one type of probiotic won’t be enough to prevent or treat RA. Prebiotics such as oligofructose and inulin provides nourishment for good bacteria in the gut. Prebiotics are bacteria that migrate through the gastrointestinal tract and into the big intestine when you eat them. These substances are not digested by the human body, but they are digested by good bacteria. Prebiotics promote the expansion and proliferation of bacteria.

## 7. Conclusions and Future Perspectives

Probiotic usage is becoming more prevalent among patients around the world, owing to a growing general knowledge of the microbiome and its role in diseases and nutrition. Throughout this review, scientists have also seen an upsurge in probiotic utilization amongst individuals with RA. In addition, the use of probiotics for 6 months did not lead in any substantial change clinically reported from RA subjects. Probiotics are microorganisms that innately inhabit the gut as the component of the microbiome and potentially be beneficial to human health. They have been utilized to support clients in restoring the microflora followed by long antibiotic or gut acid–altering pharmaceutical use. Due to their efficiency, bifidobacteria and lactobacilli are the most often utilized probiotics. There is evidence that probiotics can reduce immune cells such as fecal transplantation for *Clostridium difficile* illness [108]. 

Healthy eating/diet and medical care are at least two targets that may benefit from the use of probiotics. In addition, a probiotic supplement can help in reducing swelling and establishing a diverse microbiome with minimal negative effects. Probiotics have indeed been researched in RA and other rheumatic disorders using in vitro microbial bacterial growth studies and animal tests. Results demonstrated a reduction in the cytokines (IL-6) with the initiation of probiotics. In RA, IL-6 is a key cytokine that has been linked to joint destruction. Randomized controlled trials, on the other hand, have not found any beneficial impacts of probiotics in RA. 

Potentially, probiotics might provide a greater probability of being effective in treating diseases based on the anticipated etiology and the findings of fundamental science investigations, especially considering the consequences and results, especially in light of the implications of the microbiome actively participating in causing immunological dysregulation and increased inflammation in RA. Probiotics could be useful in assisting with the use of DMARDs. Colifant (probiotic bacterium *Escherichia coli)* has been proven to improve methotrexate treatment efficacy by lowering inflammation. Since there had been no variations in average results, few individuals have far worse consequences after taking probiotics. It is unclear whether this is attributable to probiotic usage, perhaps probiotics are introduced in response to deteriorating illness symptoms, or if the two are separate. 

A lack of knowledge about probiotic strains and dosage was and is a problem for researchers. As probiotics are often non-prescription, most people start or stop taking them without any medical/specialized advice, making it difficult to quantify exactly how long they’ve been in usage form. The microbiome is becoming increasingly important in the treatment of autoimmune. Overall, there is minimal research on the utility of probiotics in rheumatoid disease. Prospective systematic reviews would be more useful in determining the efficacy of probiotics in RA as well as which kinds provide the most benefit.
